# Effect of electric field on the electrical properties of a self-assembled perylene bisimide†

**DOI:** 10.1039/c8ra06870d

**Published:** 2018-10-04

**Authors:** Reza Saberi Moghaddam, Emily R. Draper, Claire Wilson, Hadi Heidari, Dave J. Adams

**Affiliations:** School of Chemistry, University of Glasgow Glasgow G12 8QQ UK dave.adams@glasgow.ac.uk; School of Engineering, James Watt Nanofabrication Centre, University of Glasgow Glasgow G12 8QQ UK

## Abstract

A functionalised perylene bisimide forms two different self-assembled structures in water depending on the solution pH. Structure 1 (formed at pH 6.2) consists of a fibrous structure, whilst structure 2 (formed at pH 9.4) consists of disordered aggregates. Despite being formed from the same molecule, structure 1 shows higher stability under illumination and electric field than structure 2, demonstrating that the nature of the self-assembled aggregate is critical in devices. Interestingly, both structures show p-type behaviour.

## Abstract 2

A functionalised perylene bisimide forms two different self-assembled structures in water depending on the solution pH.

## Graphical abstract

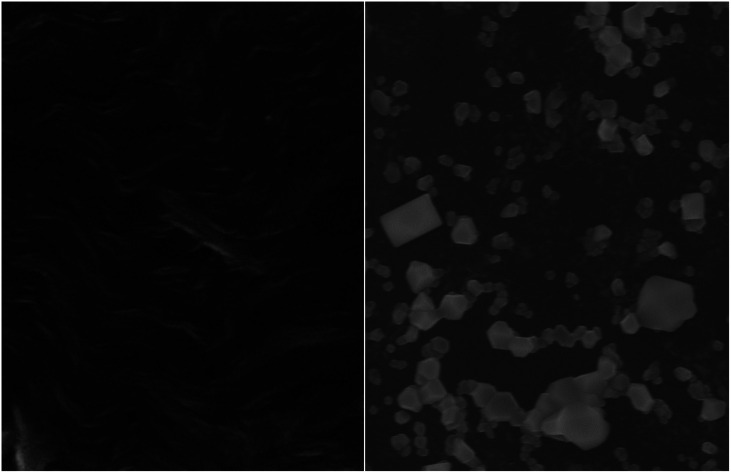

Conjugated small molecules have shown promising results in optoelectronic devices such as photovoltaics (PVs),^1^ field effect transistors (FETs),^2,3^ light emitting diodes (LEDs),^4^ and photodetectors.^5^ Perylene bisimides (PBIs, also called perylene diimides) are well-known electron transporting/accepting n-type organic semiconductors for optoelectronic devices.^6,7^ PBIs could show high electron conductivity and are the best non-fullerene n-type materials for organic photovoltaic applications.^8^ These materials also have high extinction coefficients, high thermal and chemical stability, and chemical tunability.^9,10^

PBIs can be used to form useful FETs with high electrical conductivity. The change of electrical properties of PBI is due to the different stacking of the molecules which is influenced by the structure, solvent and concentration used to self-assemble the PBIs. For example, *N*,*N*′-1*H*,1*H*-perfluorobutyl dicyanoperylenecarboxydiimide (PDIF-CN_2_) forms large grains upon post-thermal annealing of a spin-coated film at 110 °C under vacuum, resulting in efficient n-type channel field effect transistor.^11^ Jones *et al.* prepared efficient air-stable n-type FETs based on a core-cyanated PBI derivative.^12^ They showed a significantly higher conductivity for the thermally evaporated PBI derivative in a top contact configuration, while substantial drop of conductivity observed for the solution-processed PBI-based FET for a bottom gate structure.

The formation of controlled crystalline structures of PBIs to achieve high charge carrier mobility is difficult. Where the self-assembly can be controlled, this can lead to enhancement of their electrical conduction. Oh *et al.* observed solution based thin film formation of a PBI derivative in an OFET structure.^13^ The OFET device showed field effect n-type property with good electrical conductivity as a result of the slip-stacked face-to-face molecular packing of the PBI molecules and their dense parallel arrangement. Another study reported a liquid crystal (LC) PBI with space-charge limited current shows higher conductivity under ambient conditions.^14^ These LC PBIs form one-dimensional columnar stacks with intermolecular π–π orbital overlap to enhance mobility. Theoretical work reported by Delgado *et al.* showed the change of electron and hole conductivity upon addition of different end-substituted and core-substituted groups to a PBI.^15^ The change is due to different structural forms of the PBI.

PBIs structure are intrinsically insoluble and mostly used as fluorescent dyes with high fluorescent quantum yield. These materials are however well-known for their excellent n-type behaviour for different optoelectronic devices such as solar cells and field effect transistors (FETs). Water-based PBIs are promising for biofriendly optoelectronic device application with the possibility of PBI thin film formation in PVs and FETs. However, there is limited information in the literature regarding the lateral field effect conductivity of water-based perylene structure in a FET configuration at dark and under illumination.

PBIs in general can form a range of supramolecular structures, which depend on different types of intermolecular forces such as hydrogen bonding, π–π stacking and metal–ligand interactions.^10^ Among these non-covalent interactions, π–π stacking plays an important role in self-assembly of PBI derivative in both solution and films.^16,17^ The dynamics of the supramolecular structure can be controlled *via* different conditions such as the pH, temperature and concentration. PBIs can form either H- or J-type aggregates,^18–20^ although we and others have recently highlighted that this assignment needs to be completed with great care.^21,22^

We have been working with a series of amino acid functionalised PBIs. These have the advantage of being water-soluble, and we have shown that it is possible to control the aggregation type and electronic behaviour by varying the amino acid substituents.^23^ The chemical structure of the water-soluble alanine-appended PBI (PBI-A) studied in this work is shown in Fig. 1. The aggregation of PBI-A in water is driven by the hydrophobicity of the PBI core in the aqueous environment. The structures formed depend on the pH of the solution. We have previously shown for this molecule that worm-like micelles are formed at a pH of less than 7, with gels being formed by a transition to fibres below a p*K*_a_ of 5.4.^24^ The control of self-assembled structure formation of PBI-A film along with its promising photoconductivity^25,26^ makes it as an interesting candidate for the next generation semiconductor devices. Previously, we have focussed on preparing films from PBI-A in the mono-deprotonated state. Here, we specifically compare films prepared from PBI-A at two different solution pH. The degree of deprotonation is different at these two pH values, which affects the aggregation and self-assembly. We show that this directly affects the film quality. We also show that this n-type semiconductor can show p-type behaviour.

**Fig. 1:**
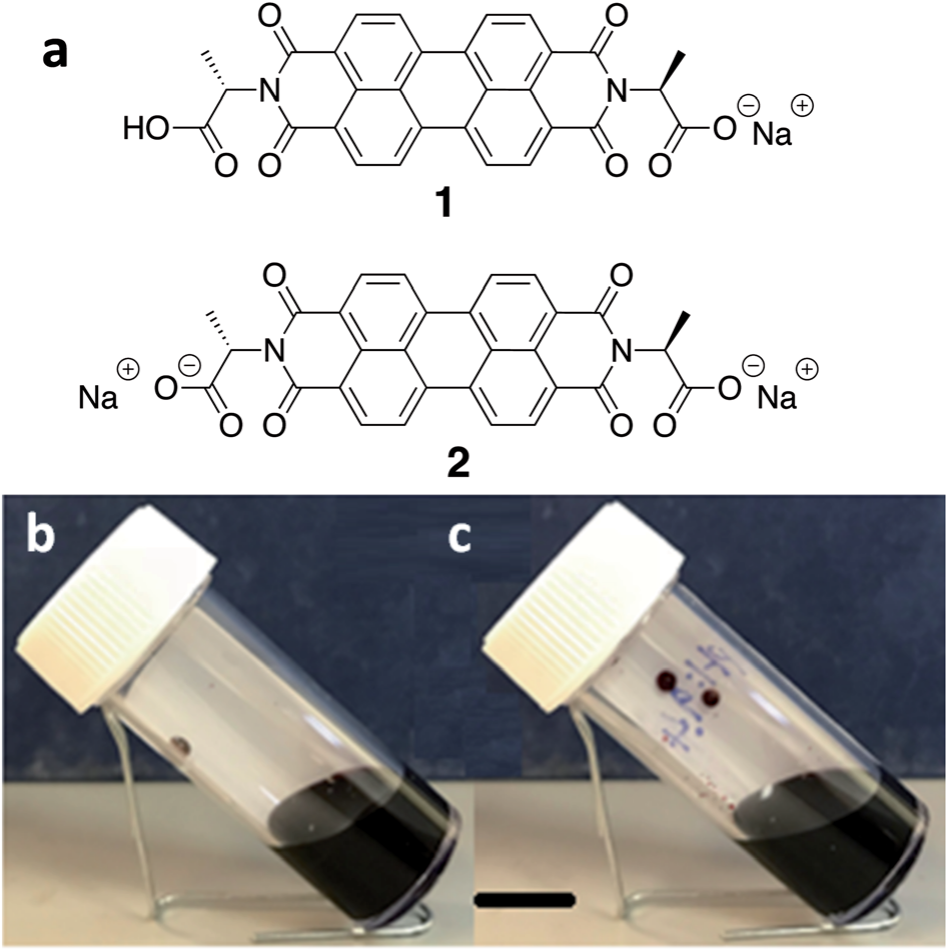
(a) Chemical structure of the alanine-functionalised perylene bisimide (PBI-A) as singly deprotonated (1) and doubly deprotonated (2) forms. (b) Photographs of solutions of the PBI at pH 6.2 (b, forming structure 1) and at pH 9.4 (c, forming structure 2). The black scale bar indicates 1 cm.

PBI-A was synthesised as described previously.^27^ This molecule has two apparent p*K*_a_.^24^ The PBI can be dispersed in water by raising the pH above the lowest p*K*_a_ of the molecule. This can be achieved by using a single equivalent of a base (formally to deprotonate a single carboxylic acid), or with two equivalents of base to form the doubly deprotonated species. Solutions of PBI-A were prepared at a concentration of 5 mg mL^−1^. On adding a single equivalent of base, the pH of this solution was 6.2. Slightly viscous solutions with a shear-thinning behaviour were formed (Fig. 1b) as can be seen the viscosity measurements (Fig. S1, ESI†). Shear thinning can be assigned to the presence of worm-like micelles as they align at high shear rates.^24^ Films can be formed from these solutions by simply drying on a surface. Long, anisotropic structures are present after drying, as shown by SEM (Fig. 2a). We refer to these as structure 1 throughout this report.

**Fig. 2:**
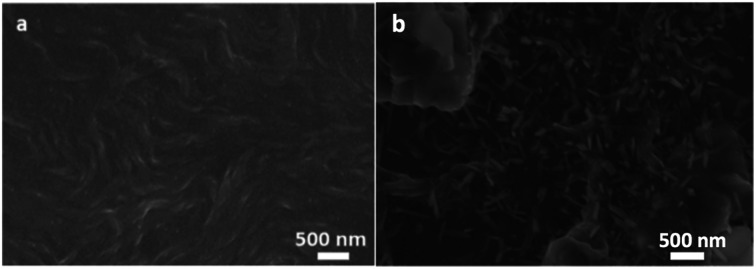
SEM images of PBI-A for (a) structure 1 and (b) structure 2 as formed by drying on a silicon substrate. The scale bar represents 500 nm in both cases.

Adding two equivalents of base results in a solution at pH 9.4. The doubly deprotonated PBI-A does not self-assemble into defined structures, resulting in a lower viscosity (Fig. S1, ESI†). We have shown previously by small angle scattering that there is limited self-assembly under these conditions.^28^ On drying, ill-defined aggregates are formed, which we refer to as structure 2 (Fig. 2b). The differences between structures 1 and 2 arise from the charge on the PBI-A, with the 2 being more negatively charged and so more soluble in water, and 1 being less charged and therefore more hydrophobic. There are slight differences in the film morphologies for both structures compared to our previous reports; this is due to us using hydrophilic surfaces here whilst our previous data used hydrophobic surfaces (comparative data are shown in Fig. S2, ESI†).

UV-Vis absorption and photoluminescence (PL) spectra of films formed from both structures are shown in Fig. 3. The absorption spectra of both films are similar, with a slightly stronger 0–0/0–1 vibronic band ratio for structure 2 as compared to 1. The different ratio of the peaks indicates different molecular packing in the structures.^9,21^

**Fig. 3:**
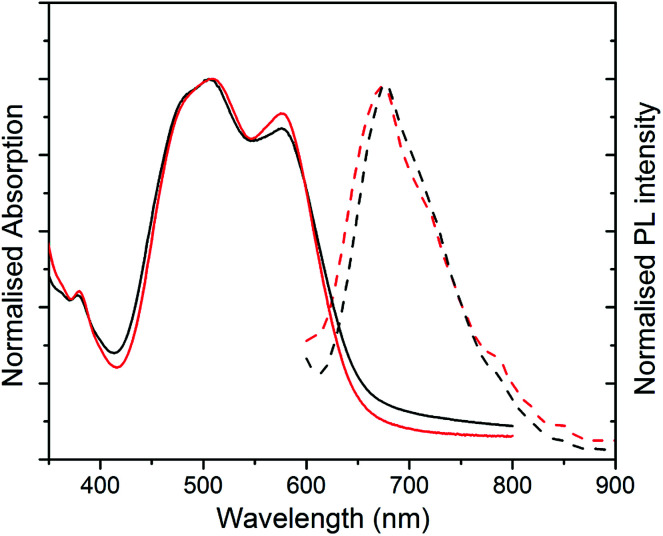
Normalised UV-Vis absorption (solid lines) to absorption peak at 505 nm and normalised PL intensity to the emission peak at 674 nm (dashed lines) for PBI-A film excited at 500 nm for structure 1 (black data) and (b) structure 2 (red data).

The PL spectra for both films (excited at 500 nm) appear similar with a more resolved shoulder at longer wavelength for structure 2, which is due to the stronger 0–0 vibronic band absorption. The non-normalised PL spectra with maximum absorption for two structures are compared in Fig. S3 (ESI†). The maximum PL peak is quenched significantly for structure 1 in comparison with structure 2. This substantial decrease in PL intensity peak can be explained as a result of fibre formation in structure 1 and a better charge separation upon photoexcitation. For structure 2, the stronger PL is an indication of amorphous structure and consequently more exciton quenching.

We prepared devices with either structure 1 or structure 2 as the active layer. The fabrication procedure for our devices is shown as a schematic diagram in Fig. 4. Briefly, p-doped Si coated by 300 nm SiO_2_ was used as a substrate and a FET device with bottom gate top contact architecture is fabricated. A PBI-A film with either structure 1 or structure 2 acted as the active layer. Gold contacts with 25 nm thicknesses were evaporated as the top source and drain contacts *via* thermal evaporation system. Using these devices, we examined the effect of electric field on the photoconductivity and structure of films comprised of either structure 1 or 2. The electrical properties of these two structures are compared in the dark and UV light illumination under different applied electric fields.

**Fig. 4:**
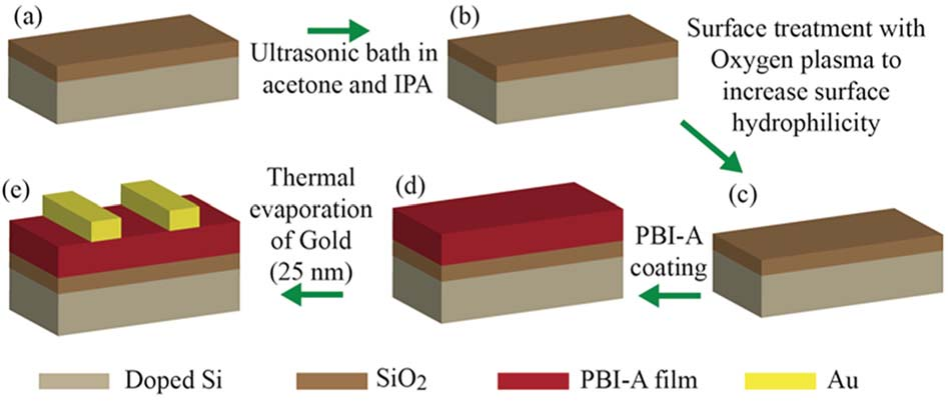
Schematic of device fabrication procedure: (a) p-doped silicon/SiO_2_, (b) ultrasonic bath in acetone and isopropanol each for 10 minutes, (c) oxygen plasma treatment at 50% power for 10 seconds, (d) drop-casting PBI-A solution on top of SiO_2_ and (e) evaporation of 25 nm gold as a source and drain contact.

The electrical conductivity of both structures was measured in the dark, and under irradiation with 365 nm light at different gate applied positive voltages of between −40 to 50 V (Fig. 5). Illumination with this wavelength was chosen on the basis of our previous reports.^9^ We observe an increase in conductivity under UV illumination, which is in agreement with our previous work^9,21,29^ and for related PBIs by other groups.^25^ This is due to the formation of radical anions and dianions, which are long-lived charged species under UV illumination and enhance the conductivity of PBI-A film.^9,30,31^ The device formed using structure 1 shows a substantial source-drain current from 160 nA in dark to 20.4 μA under UV illumination. These currents are under −40 V gate bias voltage. A weak p-type behaviour is observed for both dark and light currents. The source-drain current in dark decreases from 160 nA at −40 V to 37.5 nA at 50 V. Under 365 nm illumination, the currents for structure 1 changes from 20.4 μA at −40 V to 19 μA at 50 V. As a result, the p-type field effect transistor for structure 1 is stronger in the dark (Fig. 5a).

**Fig. 5:**
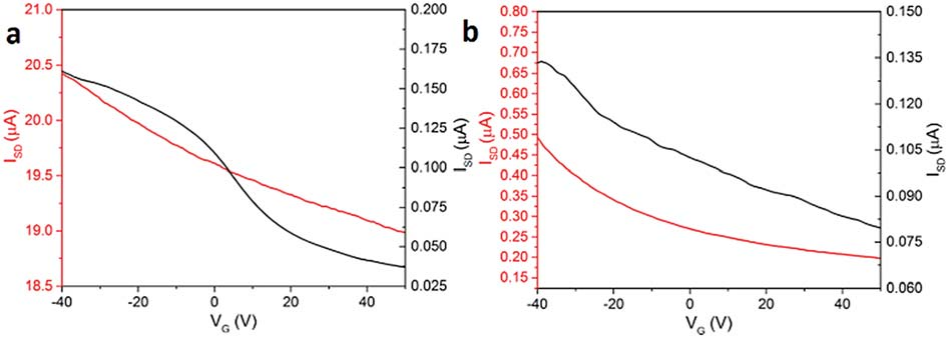
Source-drain current *versus* gate voltage for PBI-A film with (a) structure 1 and (b) structure 2 in the dark (black line) and illuminated with 365 nm light (red line).

For devices formed using structure 2, the conductivity in both the dark and under illumination also showed weak p-type behaviour. The dark current drops from 135 nA at −40 V to 80 nA at 50 V gate voltage. The conductivity under 365 nm illumination changes from 500 nA at −40 V to 200 nA at 50 V. Structure 2 shows the same p-type behaviour in both dark and under illumination as shown in Fig. 5b. This is similar to the effect observed by Besar *et al.* in OFET devices based on quaterthiophene core and the assembled peptide forming 1D nanostructures. The high off current between source-drain is due to the significant ionic current in the material due to the amino acid groups.^30^

To explain the better conductivity of structure 1 compared to structure 2, we investigated the films in the dark under an applied electric field and after the simultaneous irradiation and an applied electric field. To irradiate the films, we used a 365 nm LED as we have previously shown that there is a significant enhancement of the conductivity of a film of structure 1 under this wavelength.^9,21,29^ Under an applied electric field (gate) through the film in the dark, structure 1 does not show any significant morphological change (compare Fig. 6a with the structure shown in Fig. 2a). On application of an electric field and the LED, the films change morphology, but continuous domains can still be seen; the film shows the presence of significantly smaller fibres compared to before the application of the field and LED. These are however still connected to each other. Whilst we are unaware of other examples of changes in PBI films on application of an electric field, it is well known that electromechanical forces can cause changes in other systems.^32,33^

**Fig. 6:**
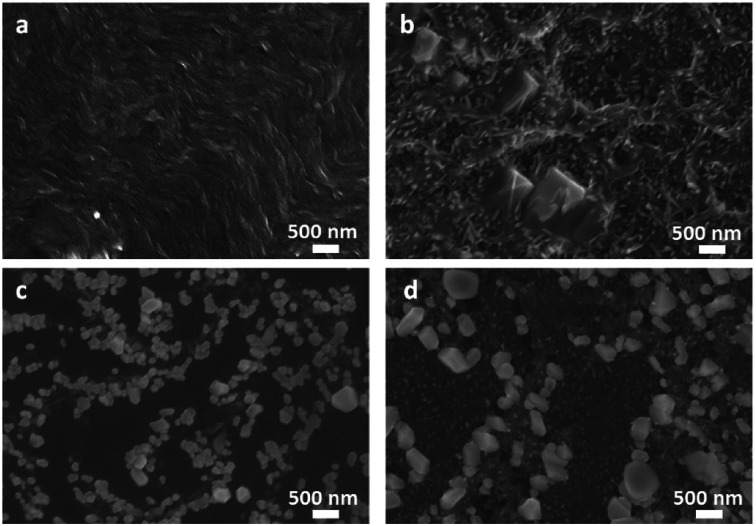
SEM image of films formed from (a) structure 1 at dark and after applied electric field; (b) structure 1 after UV and applied electric field; (c) structure 2 at dark and after applied electric field; (d) structure 2 after UV and applied electric field.

In comparison, application of the electric field to structure 2 results in the domains becoming smaller (compare Fig. 6c with 2b). The film of structure 2 under the applied electric field in the dark (Fig. 6c) shows structures with more dispersed white bright objects. As observed in the SEM image in Fig. 6d, these white features become less dispersed over the film after illumination and an applied electric field. The presence of these features could be due to the presence of sodium salts formed in structure 2. However, powder X-ray diffraction (pXRD) measurements (Fig. S4, ESI†) of films of both structure 1 and structure 2 are similar and show no peaks which could be ascribed to sodium salts. Hence, the lower film conductivity and current stability can be explained by the formation of more disordered, small aggregated domains, which importantly are not making a continuous pathway between two electrodes.

The field effect transistors based on both structure 1 and 2 show p-type behaviour in a bottom gate top contact configuration. This behaviour is not expected, as PBI is known to be an n-type material due to high electron affinity of the perylene core of PBI.^11,12^ In a recent study presented by Draper *et al.*, PBI-A showed an ionisation potential of −5.72 eV and an electron affinity of −3.91 eV.^23^ Additionally, we have significant evidence for n-type behaviour of this molecule.^23,24^ As such, the reason for the observed p-type behaviour here is unclear. A weak p-type behaviour of a peptide-functionalised, self-assembled PBI was previously observed by Eakins *et al.*^34^ Silberbush *et al.*^35^ found a substantial increase of hole transport of a peptide fibril network under various relative humidity conditions. Hence, this p-type behaviour might be due to the role of the amino acid (or peptide in the case of Eakins *et al.*^27^), or the existence of ions in the film, which modulate charge injection, and transport in PBI. Alternatively, as discussed by Delgado *et al.*,^15^ the presence of functional group in a PBI can result in a lowering of the re-organisational energy of holes and consequently improved hole conductivity. It may be that the molecular packing on drying on the surfaces here leads to suitable morphological changes that favour p-type behaviour. Finally, we note that recent work by Zhang *et al.* have suggested that PBI films can show either p-type or n-type behaviour depending on the ratio of dianion to radical anion in the film.^36^ This behaviour is the subject of further investigation.

In conclusion, a water-dispersible perylene bisimide can form different structures depending upon the absolute solution pH. In a bottom gate top contact FET configuration, this material shows p-type behaviour with a substantial increase of current under 365 nm UV illumination. The ambipolar behaviour of water-based perylene bisimide derivative under different processing conditions may provide a route toward developing ambipolar FET devices based on the single material.

## Conflicts of interest

There are no conflicts to declare.

## Supplementary Material

RA-008-C8RA06870D-s001
