# Supplementary material for: Effect of electric field on the electrical properties of a self-assembled perylene bisimide
Source: RSC Adv. 2018 Oct 4;8(59):34121–5. doi: 10.1039/c8ra06870d (PMC9086733; doi:10.1039/c8ra06870d)
Supplement: RA-008-C8RA06870D-s001 [file RA-008-C8RA06870D-s001.pdf]

Electronic Supplementary Material (ESI) for RSC Advances.
This journal is © The Royal Society of Chemistry 2018

Effect of electric field on electrical properties of a self-assembled perylene bisimide

Reza Saberi Moghaddam,[a] Emily R. Draper,[a] Claire Wilson,[a] Hadi Heidari,[b] and Dave J. Adams*[a]

[a] School of Chemistry, University of Glasgow, Glasgow, G12 8QQ, UK.
[b] School of Engineering, University of Glasgow, Glasgow, G12 8QQ, UK.

Email: dave.adams@glasgow.ac.uk

Supporting Information

Procedures and Instruments

Solution preparation: PBI-A was synthesised as reported previously.[1] A solution of the PBI-A (5 mg/mL) was prepared by adding water and adding either one (for structure **1**) or two (structure **2**) molar equivalents of a solution of NaOH (0.1 M). The dispersion was stirred overnight leading to complete dissolution. The pH was measured at lab temperature (22-23˚C) and recorded prior to device fabrication.

pH measurements: A FC200 pH probe (HANNA instruments) with a 6 mm × 10 mm conical tip was used for all pH measurements. The accuracy of pH probe was ±0.1.

UV-vis absorption measurement: Samples for spectroscopy were prepared on microscope slides. Before use, the microscope slides were cleaned with acetone, and isopropanol for 10 minutes in an ultrasonic bath each, followed by oxygen plasma for 10 minutes. The film was formed via drop casting of the required PBI-A solution. The UV-vis absorption spectroscopy measurement was obtained via using Agilent Cary 60 UV-Vis.

Device fabrication: Devices were fabricated on clean silicon substrate. Silicon substrates used in this study were as p-doped silicon covered by thermally grown 300 nm SiO_2. After cleaning silicon substrate in acetone and isopropanol using an ultrasonic bath for 10 minutes each, the samples were treated in a Diener Oxygen Plasma unit for 10 seconds at 50 % power in order to make the Si/SiO_2 surface more hydrophilic. Then, 2.5 µL of 5mg/mL PBI-A solution with either structure **1** or **2** was drop-casted over 0.5 cm × 0.5 cm Si/SiO_2 surface area. After film formation, 25 nm gold with the rate of 0.25-0.3 nm/s at pressure of 2×10^{-6} mbar was evaporated as a top source and drain contact via shadow mask.

Scanning microscopy (SEM): p-doped Si substrates coated with SiO_2 were used for the SEM measurement. The silicon substrates cut to 0.5 cm × 0.5 cm were cleaned with acetone and isopropanol each for 10 minutes in ultrasonic bath. For the hydrophilic silicon substrate, the cleaning procedure continued with 10 seconds oxygen plasma at 50 % power. Then, 2.5 µL of 5mg/mL of the required PBI-A solution was drop-cast on to the surface and allowed to dry. Samples were prepared on silicon substrate with p-doped silicon covered by thermally grown 300 nm SiO_2. The samples were imaged uncoated. The SEM instrument was used in this study was SU8240 ultra-high resolution scanning electron microscope. The SEM was operated at 5 kV and 10 kV, with a working distance of 8 cm.

Device characterisation: Devices were measured following device fabrication. The measurements were carried out at ambient condition with Keysight B1500a. The transfer curve was measured with 50 V fixed voltage between source and drain while the gate voltage varies from 0 V to 60 V. The leakage current was less than 100 nA. The devices were irradiated with a 365 nm wavelength LED with a power of 2.3W, with an irradiation time of 20 minutes.

XRD measurements: Measurements were carried out on a Panalytical X'pert Pro multipurpose diffractometer with a Cu Kα1 source (λ = 1.5407 Å). Patterns were measured between 0 to 60° 2θ for 2 hr (step size 0.033°, time per step 295.3 s, and scan speed 0.014°/s). Samples for XRD are prepared on zero background Si substrate. PBI-A solution (5 mg/mL) with 1 or 2 equivalents of 0.1 M NaOH (i.e. structure **1** or structure **2**) was drop casted on the substrate and dried to form a film. To form a thin layer, 10 µL of PBI-A solution drop casted on the substrate. For the thick layer, 1 ml of PBI-A solution drop casted on top of the substrate.

Photoluminescence (PL) measurement: The PL measurements were performed on an Agilent Cary Eclipse Fluorescence spectrometer. Emission spectra were collected between 530 nm and 900 nm with 500 nm excitation wavelengths.

Rheological measurements: Dynamic viscosity measurements were performed by Anton Paar Physica MCR101 rheometer. A cone and plate measuring system was used to perform viscosity measurements. A 75 mm diameter plate with a 1° cone angle and flat plate system was used for the measurements. 2 mL of the solution was transferred to the plate for the viscosity measurement. Experiments were performed at 25 ˚C. Two repeats were carried out for each measurement.

Supplementary Figures

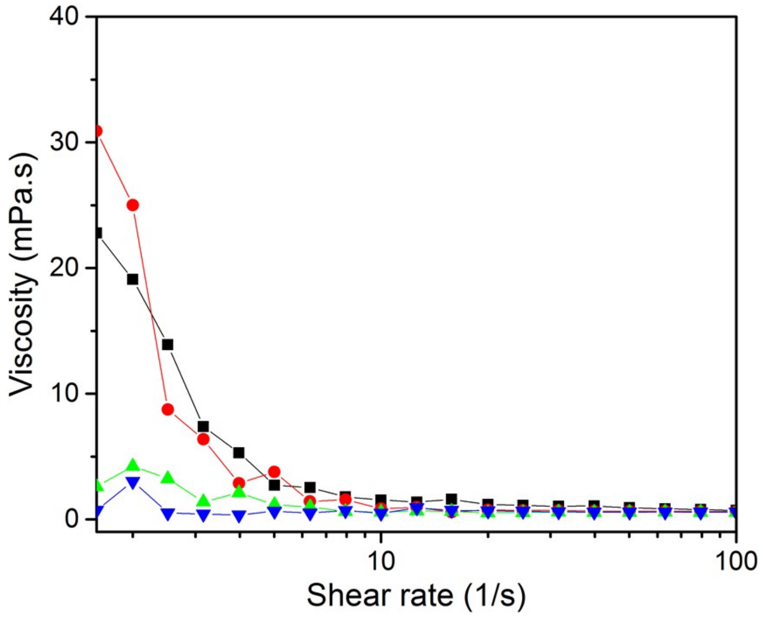

Figure S1. Dynamic viscosity measurements for PBI-A solution of structure **1** (black and red) and structure **2** (green and blue) at a concentration of 5 mg/mL.

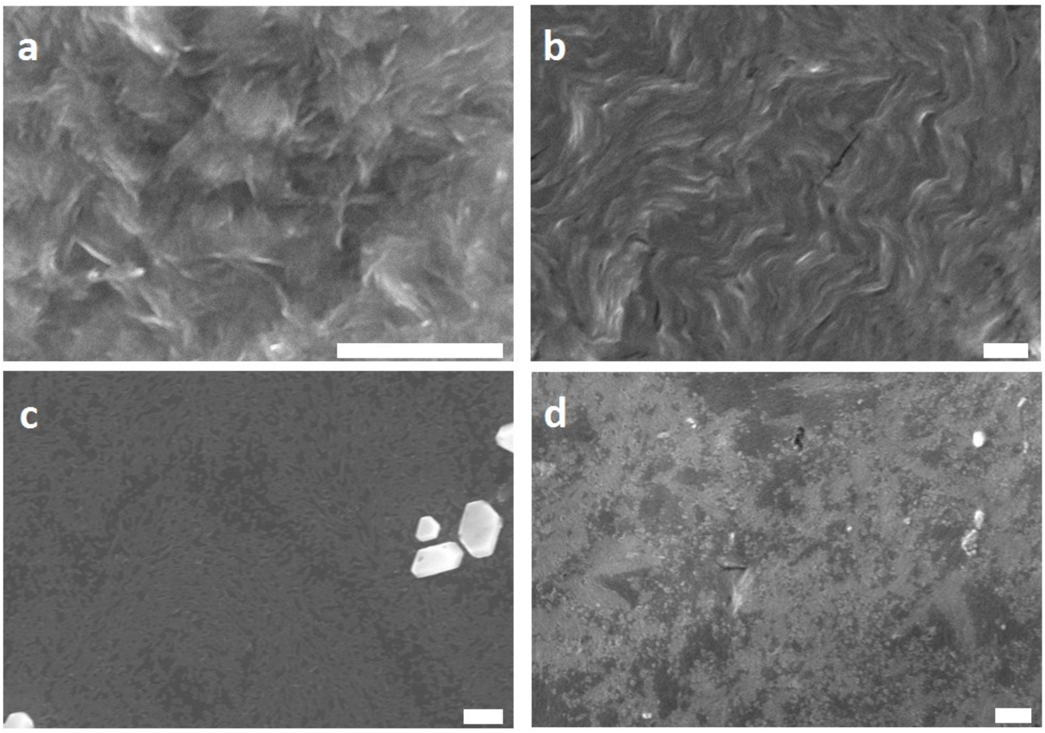

Figure S2. Structure **1** on (a) hydrophilic and (b) hydrophobic silicon substrate. Structure **2** on (c) hydrophilic and (d) hydrophobic silicon substrate**.** The scale bar is 1μm.

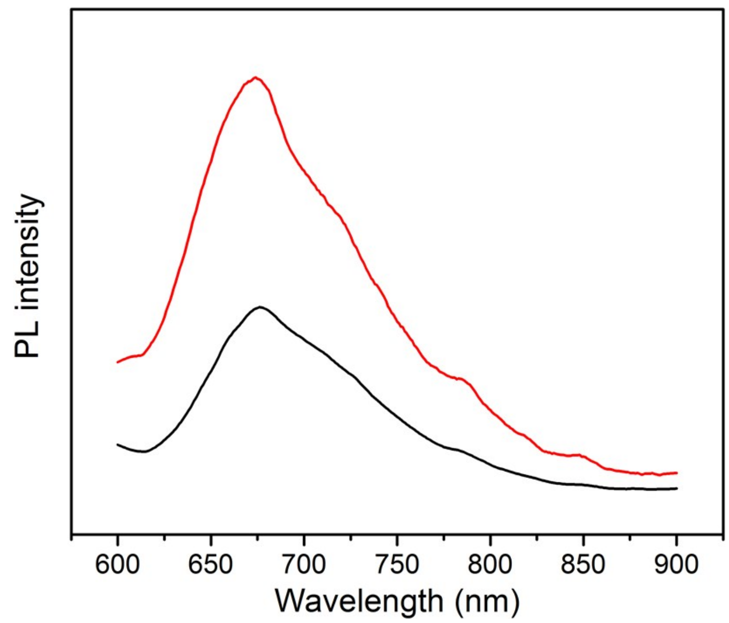

Figure S3. PL intensity comparison of PBI-A film with structure **1** (black) and structure **2** (red).

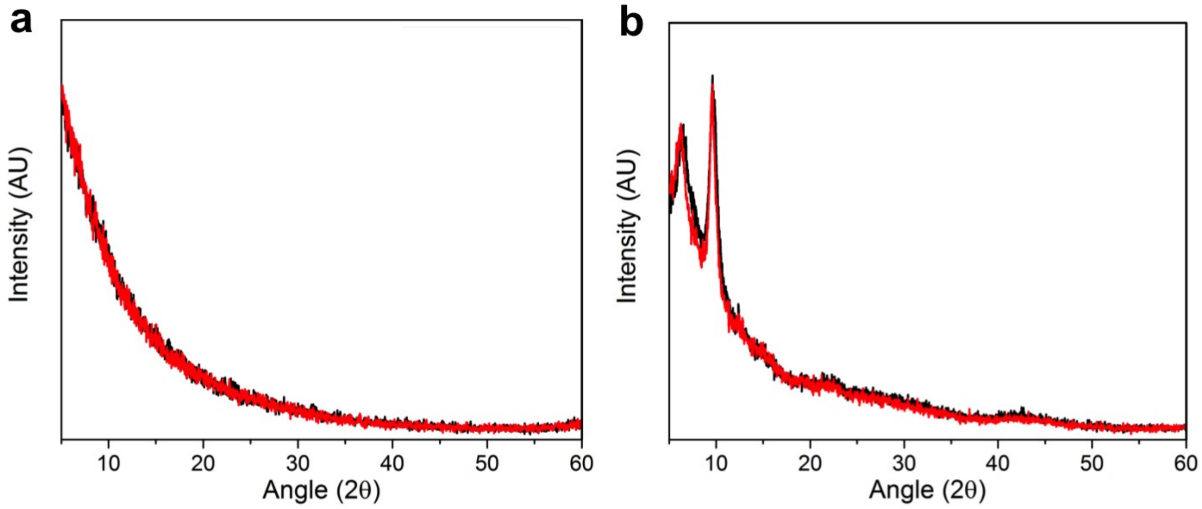

Figure S4. Comparison of XRD patterns from measurements of structure **1** (black data) and structure **2** (red data) for (a) thin films and (b) thick film

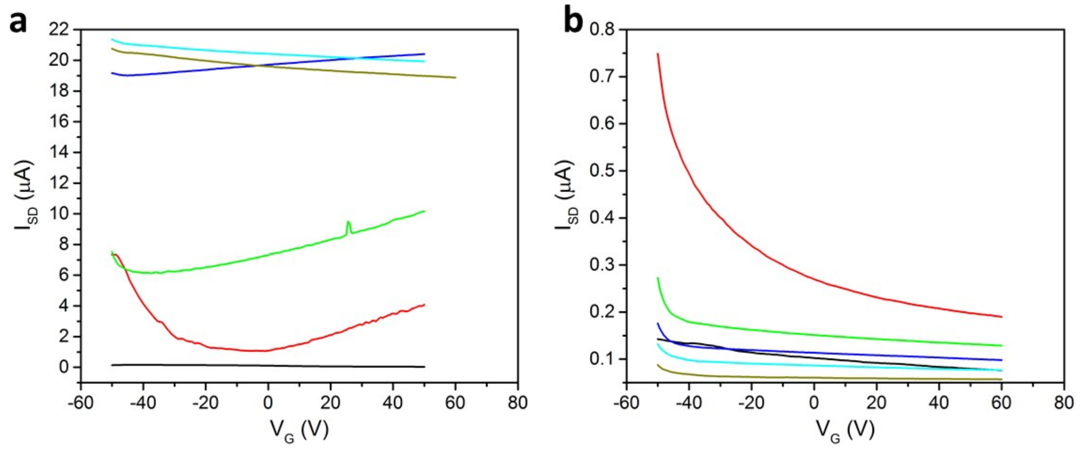

Figure S5. Source-drain current vs gate voltage for (a) structure **1** and (b) structure **2** at different illumination times. Dark current (black), under 365 nm LED illumination: first scan (red), second scan (green), after 10 min (blue), after 14 min (cyan) and after 20 min (dark yellow).

References

1. E. R. Draper, J. J. Walsh, T. O. McDonald, M. A. Zwijnenburg, P. J. Cameron, A. J. Cowan and D. J. Adams, *J. Mater. Chem. C*, 2014, **2**, 5570-5575.
